# Immune life history, vaccination, and the dynamics of SARS-CoV-2 over the next 5 years

**DOI:** 10.1126/science.abd7343

**Published:** 2020-09-21

**Authors:** Chadi M. Saad-Roy, Caroline E. Wagner, Rachel E. Baker, Sinead E. Morris, Jeremy Farrar, Andrea L. Graham, Simon A. Levin, Michael J. Mina, C. Jessica E. Metcalf, Bryan T. Grenfell

**Affiliations:** 1 Lewis-Sigler Institute for Integrative Genomics, Princeton University, Princeton, NJ 08540, USA.; 2 Department of Ecology and Evolutionary Biology, Princeton University, Princeton, NJ 08544, USA.; 3 Princeton Environmental Institute, Princeton University, Princeton, NJ 08544, USA.; 4 Department of Bioengineering, McGill University, Montreal, Quebec H3A 0C3, Canada.; 5 Department of Pathology and Cell Biology, Columbia University Medical Center, New York, NY 10032, USA.; 6 Wellcome Trust, London, UK.; 7 Departments of Epidemiology and Immunology and Infectious Diseases, Harvard School of Public Health, Boston, MA 02115, USA.; 8 Princeton School of Public and International Affairs, Princeton University, Princeton, NJ 08544, USA.; 9 Fogarty International Center, National Institutes of Health, Bethesda, MD 20892, USA.

## Abstract

The future trajectory of the coronavirus disease 2019 (COVID-19) pandemic hinges on the dynamics of adaptive immunity against severe acute respiratory syndrome coronavirus 2 (SARS-CoV-2); however, salient features of the immune response elicited by natural infection or vaccination are still uncertain. We use simple epidemiological models to explore estimates for the magnitude and timing of future COVID-19 cases, given different assumptions regarding the protective efficacy and duration of the adaptive immune response to SARS-CoV-2, as well as its interaction with vaccines and nonpharmaceutical interventions. We find that variations in the immune response to primary SARS-CoV-2 infections and a potential vaccine can lead to markedly different immune landscapes and burdens of critically severe cases, ranging from sustained epidemics to near elimination. Our findings illustrate likely complexities in future COVID-19 dynamics and highlight the importance of immunological characterization beyond the measurement of active infections for adequately projecting the immune landscape generated by SARS-CoV-2 infections.

## Imperfect future immunity

Humans are infected by several seasonal and cross-reacting coronaviruses. None provokes fully protective immunity, and repeat infections are the norm. Vaccines tend to be less efficient than natural infections at provoking immunity, and there are risks of adverse cross-reactions. Saad-Roy *et al.* used a series of simple models for a variety of immune scenarios to envisage immunological futures for severe acute respiratory syndrome coronavirus 2 (SARS-CoV-2) with and without vaccines. The model outcomes show that our imperfect knowledge about the imperfect coronavirus immune landscape can give rise to diverging scenarios ranging from recurring severe epidemics to elimination. It is critical that we accurately characterize immune responses to SARS-CoV-2 for translation into managing disease control.

*Science*, this issue p. 811

## Teaser

Modeling shows how essential it is to improve precise understanding of immune responses to SARS-CoV-2.

The novel severe acute respiratory syndrome coronavirus 2 (SARS-CoV-2) betacoronavirus (β-CoV) pandemic has resulted in substantial morbidity and mortality, with over 27 million confirmed cases worldwide at the time of writing. To curb viral transmission, nonpharmaceutical interventions (NPIs), including business and school closures, restrictions on movement, and total lockdowns, have been implemented to various degrees around the world. Major efforts to develop effective vaccines and antivirals are ongoing.

Understanding the future trajectory of this disease requires knowledge of the population-level landscape of immunity, generated by the life histories of SARS-CoV-2 infection or vaccination among individual hosts. We show that the nature of secondary infection, particularly the degree of acquisition, retransmission, and clinical severity of subsequent infections with the same pathogen, is particularly important. The nature of acquired immune responses after natural infection varies substantially among pathogens. At one end of this immune spectrum, natural infection with measles (*1*) or smallpox (*2*) virus results in lifelong protection from the reacquisition and retransmission of secondary infections. Many other infections [e.g., influenza (*3*) and respiratory syncytial virus (RSV) (*4*)] confer imperfect or transient clinical and transmission-blocking immunity by either pathogen evolution or waning immunological memory. Finally, phenomena such as antibody-dependent enhancement (ADE) associated with prior natural infection [e.g., dengue (*5*)] or a vaccine [e.g., RSV (*6*)] could result in more clinically severe secondary infections. Furthermore, the immunity conferred by vaccines may not provide complete protection against reinfection and/or disease (*7*), and this protection may be inferior to that acquired after natural infection (*8*). Nevertheless, imperfect vaccines that reduce both the clinical severity and transmissibility of subsequent infections (if they do occur) can still provide population-level disease protection (*7*, *9*, *10*).

The nature of the immune response after natural SARS-CoV-2 infection remains an area of active investigation (*11*–*18*). Reports from serological population- and individual-level studies demonstrate that detectable antibody levels can wane over the first few months postinfection (*19*), yet recent findings demonstrate robust antibody responses 4 months after infection (*20*). This is broadly consistent with serum antibody levels against the seasonal coronavirus human coronavirus OC43 (HCoV-OC43) [which belongs to the same β-CoV genus as SARS-CoV-2 (*21*)], which wane on the time scale of a few months (*22*) to 1 year (*23*). Such seasonal β-CoVs (which also include HCoV-HKU1) are thought to cause repeated infections throughout life (*24*), although a significant biennial component in their dynamics implies at least some herd protection (*21*, *25*). This genus also contains other viruses that cause severe infections in humans, including Middle East respiratory syndrome and SARS-CoV-1 coronaviruses (*21*). Whereas humoral immunity to SARS-CoV-1 is believed to last up to 2 to 3 years (*26*, *27*), antigen-specific T cells against this virus were found to be detectable for at least 11 years after infection (*28*). Indeed, T cell–mediated responses likely play a central role in controlling SARS-CoV-2 replication and disease (*14*, *15*). Recent evidence of preexisting T cells (*14*, *15*) and antibodies (*29*) capable of cross-reacting with SARS-CoV-2 suggests that immunological memory responses elicited during infection with seasonal coronaviruses may also affect coronavirus disease 2019 (COVID-19) susceptibility and disease risk. Finally, although it is currently unclear whether ADE influences the pathogenesis of SARS-CoV-2, it has been hypothesized that severe COVID-19 cases may arise from the presence of nonneutralizing antibodies from prior coronavirus infections (*30*), in agreement with earlier proposals for related coronaviruses (*31*–*33*).

Various epidemiological models have been developed to capture how the diversity or variation in immune responses influences population-level infection dynamics. For instance, the well-known Susceptible-Infected-Recovered (SIR) model is suitable for modeling the dynamics of perfectly immunizing infections such as measles (*34*), whereas the Susceptible-Infected-Recovered-Susceptible (SIRS) model captures the epidemiology of imperfectly immunizing infections such as influenza; here, individuals eventually return to a fully or substantially susceptible class after a finite period of immunity, because of either waning memory or pathogen evolution (*35*). More complex compartmental models have also been developed to study infections characterized by intermediate immune responses lying between these two extremes, such as rotavirus (*36*) and RSV (*4*).

**Fig. 1:**
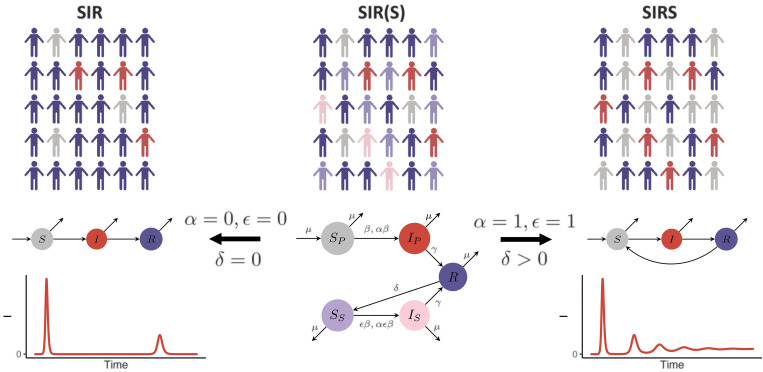
Schematic of the SIR(S) model with a flowchart depicting flows between immune classes. Here, *S*_P_ denotes fully susceptible individuals; *I*_P_ denotes individuals with primary infection that transmit at rate β; *R* denotes fully immune individuals (a result of recovery from either primary or secondary infection); *S*_S_ denotes individuals whose immunity has waned at rate δ and are now again susceptible to infection, with relative susceptibility ε; *I*_S_ denotes individuals with secondary infection that transmit at a reduced rate αβ; and μ denotes the birth rate (*37*). Illustrations and flowcharts of the limiting SIR and SIRS models are also shown (where individuals are either fully susceptible (*S*), infected (*I*), or fully immune (*R*)), along with a representative time series for the number of infections in each scenario. The population schematics were made through use of (*62*).

Here, we adopt a generalization of these models, the SIR(S) model (*35*), outlined schematically in Fig. 1 and fig. S1, to explore how the pandemic trajectory might unfold for different assumptions regarding the nature of the adaptive immune response to SARS-CoV-2 infection. Because different adaptive immune responses may be associated with variations in the proportion of severe secondary cases, we also consider a range of values for this fraction in order to explore the potential future clinical burden of SARS-CoV-2 infections. The model assumes different infection and immune phenotypes, depending on exposure history [see (*37*) for the full mathematical details]. Specifically, it interpolates between the fully immunizing SIR model, when immunity is lifelong, and the imperfectly immunizing SIRS model via the degree of susceptibility to and transmissibility of secondary infections (quantified by the parameters ε and α, respectively). As shown in the representative time series of Fig. 1, the SIR model results in recurrent epidemics fueled by births following the pandemic peak; by contrast, the SIRS model typically generates shorter interepidemic periods owing to the possibility of reinfection and the buffering of the fully susceptible birth cohort by partially immune individuals (*35*).

We begin by characterizing the effect of temporal changes in the transmission rate brought about by climate and the deployment of NPIs on the predictions of the SIR(S) model under a range of immunity assumptions. Next, we examine the impact of a transmission-reducing vaccine of varying efficacy relative to natural immunity. Finally, we estimate the postpandemic immunity landscape and clinical case burden for different possible futures (*38*) shaped by the various aspects of SARS-CoV-2 biology as well as the presence or absence of these external drivers and interventions, as well as vaccine refusal. To focus on the dynamic impact of natural and vaccinal immunity, we begin with a simple homogeneous model, which averages across known heterogeneities in COVID-19 transmission and severity [age (*39*), superspreading events (*40*), etc.]. We then use heterogeneous model extensions to show that such heterogeneities do not impact our exploration of qualitative medium-term dynamics under different immunological scenarios.

## Seasonal transmission rates and the deployment of NPIs

**Fig. 2:**
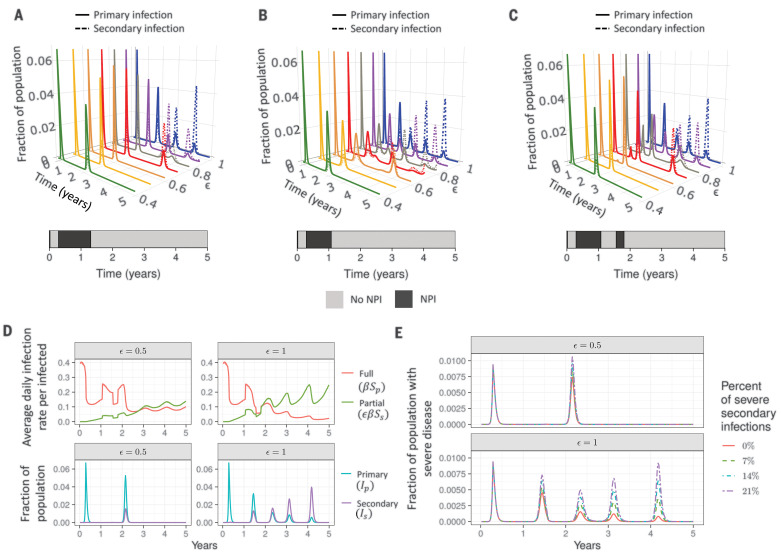
Seasonality in transmission rates and NPIs modulate disease dynamics. (**A** to **C**) Effect of NPI adoption on the time series of primary (solid lines) and secondary (dashed lines) infections with a seasonal transmission rate derived from the climate of New York City with no lag between seasonality and epidemic onset. NPIs that reduce the transmission rate to 60% of the estimated climate value are assumed to be adopted during weeks 16 to 67 (A), weeks 16 to 55 (B), or weeks 16 to 55 as well as weeks 82 to 93 (C). Colors denote individual time courses for different values of ε. (**D**) Time series of the average daily infection rate per infected individual of fully susceptible (red line) and partially susceptible (green line) individuals (top row) and the fraction of the population that is infected with primary (blue line) and secondary (purple line) infections (bottom row), for ε = 0.5 (left column) and ε = 1 (right column) for the NPI scenario outlined in (C). (**E**) Time series of estimated numbers of severe infections for the NPI scenario defined in (C) for four different estimates of the fraction of severe cases during primary infections (*x*_sev,p_) and secondary infections (*x*_sev,s_) with ε = 0.5 (top row) and ε = 1 (bottom row). These are *x*_sev,p_ = 0.14, *x*_sev,s_ = 0 (solid red line); *x*_sev,p_ = 0.14, *x*_sev,s_ = 0.07 (dashed green line); *x*_sev,p_ = 0.14, *x*_sev,s_ = 0.14 (dashed and dotted blue line); and *x*_sev,p_ = 0.14, *x*_sev,s_ = 0.21 (purple line with long and short dashes). In all panels, the relative transmissibility of secondary infections and duration of natural immunity are taken to be α = 1 and 1*/*δ = 1 year, respectively. The effects of NPIs and other parameter variations can be explored interactively at https://grenfelllab.princeton.edu/sarscov2dynamicsplots.

Medium-term dynamics will be shaped by changes in the magnitude of transmission. To explore the effect of NPIs, we considered two different scenarios for timed reductions in the force of infection to 60% of its original value [in agreement with intermediate levels of social distancing in (*21*)]. In Fig. 2, A to C, we show the time courses of primary and secondary infections, assuming single periods of NPI lasting from weeks 16 to 67 (Fig. 2A) or 16 to 55 (Fig. 2B) and two shorter periods during weeks 16 to 55 and weeks 82 to 93 separated by normal interactions (Fig. 2C). We further assume a seasonal transmission rate derived from the climate of New York City (*37*), although in principle this seasonality could also be derived from other nonclimate factors (*25*). The weekly reproduction numbers corresponding to these three scenarios are shown in fig. S2, D to F. Although these reproduction numbers are based on those obtained for the related β-CoV HCoV-HKU1 and are in general lower than those estimated during the early stages of the SARS-CoV-2 pandemic (*41*), they may be more appropriate for considering the longer-term transmission dynamics.

We find that decreases in the susceptibility to secondary infection, ε, can delay secondary peaks (compare individual time courses for different values of ε in Fig. 2, A to C). However, delayed peaks may then be larger, because of susceptible accumulation (through demography or immune waning) and dynamic resonance. These nonmonotonicities in the timing and size of secondary peaks also occur with climate-driven seasonal transmission in the absence of NPIs (*37*), and the trends are qualitatively similar if NPIs are assumed to be relaxed more gradually (fig. S11). Notably, the delay that social distancing may cause in the timing of the secondary peak can also allow for further accumulation of fully susceptible individuals. This is illustrated in the top panels of Fig. 2D, where the average infection rate per infected individual for fully (β*S*_P_; red curve) and partially (εβ*S*_S_; green curve) susceptible individuals for the social distancing scenario outlined in Fig. 2C are shown. We contrast a reduction in susceptibility to secondary infection of 50% (ε = 0.5, left panels) with no reduction in susceptibility to secondary infection (ε = 1, right panels). The corresponding fraction of primary (blue) and secondary (purple) cases are presented in the bottom panels. As can be seen, when the secondary peak does occur, the decrease in susceptibility to secondary infection (ε *<* 1), considered in the left panels, results in a greater number of primary infections during the second peak relative to the panels on the right, where ε is 1 [and the secondary infection rate per case (green curves) rises sharply].

Next, an essential part of the planning and management of future SARS-CoV-2 infections is the ability to characterize the magnitude and timing of severe cases requiring hospitalization. In Fig. 2E we consider four possible scenarios for the fraction of severe secondary cases, *x*_sev,s_ (*37*), on the basis of the scenario depicted in Fig. 2C and assuming 14% of primary cases are severe (*42*): (i) no severe cases associated with secondary infection (*x*_sev,s_ = 0; solid red line); (ii) a reduced number of severe cases with secondary infection relative to primary infection (*x*_sev,s_ = 0.07; dashed green line); (iii) comparable proportions of severe cases (*x*_sev,s_ = 0.14; dashed-dotted blue line); and (iv) a hypothetical greater proportion of severe cases with secondary infection (*x*_sev,s_ = 0.21; purple line with short and long dashes), possibly owing to phenomena such as ADE. When the assumed fraction of severe subsequent infections is high, the fraction of the population with severe infections during subsequent infection peaks is found to be comparable to or even to slightly exceed that observed during the initial pandemic peak (Fig. 2E). As the proportion of secondary infections increases during the later stages of the pandemic, these findings stress that clinical epidemiological studies of repeat infections will be critical for proper planning of health care systems. We also do not consider any long-term clinical impact of infection here (*43*). The impact of increases in clinical severity with age is addressed below.

## Vaccination

**Fig. 3:**
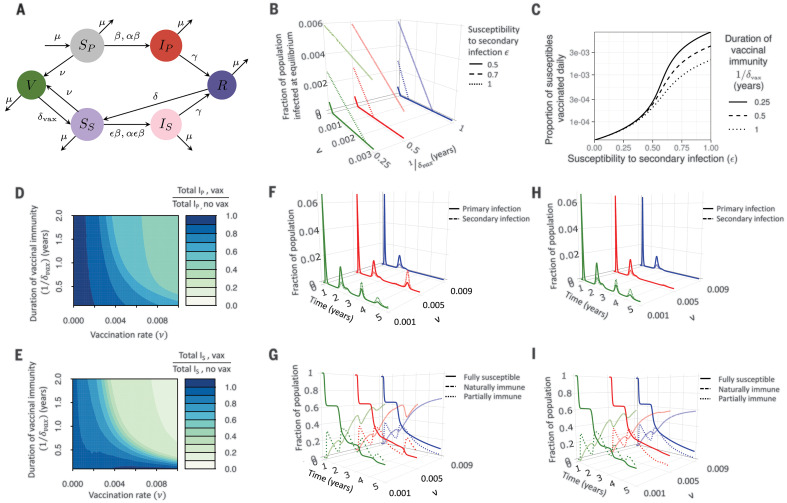
Impact of vaccination and vaccinal immunity on disease dynamics. (**A**) Modified model flowchart that incorporates a vaccinated class *V* (*37*). (**B**) Total infected fraction of the population at equilibrium as a function of the vaccination rate ν for different values of the duration of vaccinal immunity (1*/*δ_vax_ = 0.25 years, green lines; 1*/*δ_vax_ = 0.5 years, red lines; and 1*/*δ_vax_ = 1 year, blue lines) and the susceptibility to secondary infection (ε = 0.5, solid lines; ε = 0.7, dashed lines; and ε = 1, dotted lines). (**C**) Daily proportion of susceptibles who must be vaccinated in order to achieve a disease-free state at equilibrium as a function of ε for different values of the duration of vaccinal immunity (1*/*δ_vax_ = 0.25 years, solid line; 1*/*δ_vax_ = 0.5 years, dashed line; and 1*/*δ_vax_ = 1 year, dotted line). In (B) and (C), the relative transmissibility of secondary infections and duration of natural immunity are taken to be α = 1 and 1*/*δ = 1 year, respectively, and the transmission rate is derived from the mean value of seasonal New York City–based weekly reproduction numbers (${\bar{R}}_{0}$ = 1.75) (fig. S2C) (*37*). (**D** and **E**) The ratio of the total number of primary (D) and secondary (E) infections with vaccination versus without vaccination, during years 1.5 to 5 (i.e., after the introduction of the vaccine) are plotted as a function of the weekly vaccination rate ν and the duration of vaccinal immunity 1*/*δ_vax_. (**F** to **I**) Time series of the various immune classes plotted for different values of the vaccination rate ν. The top row [(F) and (H)] contains the time series of primary (*I*_P_, solid lines) and secondary (*I*_S_, dashed lines) infections, whereas the bottom row [(G) and (I)] contains the time series of the fully susceptible (*S*_P_, solid lines), naturally immune (*R*, dashed lines), and partially immune (*S*_S_, dotted lines) subpopulations. The duration of vaccinal immunity is taken to be 1*/*δ_vax_ = 0.5 years (shorter than natural immunity) in (F) and (G), and 1*/*δ_vax_ = 1 year (equal to natural immunity) in (H) and (I). In (D) to (I), the relative susceptibility to secondary infection, relative transmissibility of secondary infections, and duration of natural immunity are taken to be ε = 0.7, α = 1, and 1*/*δ = 1 year, respectively. Vaccination is introduced 1.5 years after the onset of the epidemic (i.e., during the 79th week) following a 40-week period of social distancing during which the force of infection was reduced to 60% of its original value during weeks 16 to 55 (i.e., the scenario described in Fig. 2B), and a seasonal transmission rate derived from the climate of New York City with no lag is assumed.

The availability of an effective vaccine would be a key intervention against SARS-CoV-2, and numerous candidates are in development (*44*, *45*). Intuitively, if the effective vaccination rate is sufficiently high, then vaccinal herd immunity generated by a transmission-blocking vaccine could control or eliminate the infection. However, this becomes harder to achieve when vaccinal and natural immunity is imperfect and secondary infections occur, or when logistical or other constraints limit vaccine deployment. We extend the model (*37*) to include a vaccinated class, *V*, and make the relatively optimistic assumption that a transmission-reducing vaccine begins to be introduced to general populations (the *t*_vax_) after 1.5 years. We also consider seasonal transmission rates, as in fig. S3, and the deployment of NPIs according to the scenario described in Fig. 2B. We assume that a constant proportion, ν, ranging from 0% ≤ ν ≤ 1% of the fully and partially susceptible populations (*S*_P_ and *S*_S_), is effectively vaccinated every week and acquires transmission-blocking immunity for, on average, a period 1*/*δ_vax_. For comparison, it was estimated that in response to the 2009 H1N1 pandemic, one or more doses of the monovalent vaccine were administered to 80.8 million vaccinees during October 2009 to May 2010 in the United States (*46*), which implies a rate of vaccination coverage of about 27% after a period of 8 months for persons aged at least 6 months in the United States, although rates between different nations varied (*47*). This crudely corresponds to a weekly vaccination rate of 1% (*48*) (as with other parameter variations, different scenarios for vaccination can be explored with the accompanying Shiny application). Finally, we assume that the immunity conferred from effective vaccination wanes at rate δ_vax_, which in general may differ from the waning rate of immunity from natural infection, δ. The modified set of ordinary differential equations in this scenario corresponding to the flowchart in Fig. 3A is also presented in (*37*).

In Fig. 3B, we begin by considering the long-term equilibrium infection burden (*37*) driven by vaccination at a weekly rate ν, for a variety of immunity assumptions. As expected, a reduction in the susceptibility to secondary infections (ε) results in a smaller number of infections at steady state in the absence of vaccination. Further, both ε and the duration of vaccinal immunity (1*/*δ_vax_) affect the vaccination rate required to achieve a disease-free state at equilibrium. At the limit of fully immunizing primary infections and vaccines (ε = 0), relatively low vaccination rates are sufficient to achieve zero infections at steady state. However, as immunity becomes more imperfect (larger ε), increasingly high vaccination rates are required to eliminate infections, particularly when the duration of vaccinal immunity is short. This is further emphasized in Fig. 3C, where the minimum vaccination rate ν required to achieve a disease-free state at equilibrium (*37*) is shown as a function of ε for different values of the duration of vaccinal immunity. These results underline that reductions in infection achievable through vaccination are inherently related to the efficacy of the vaccine and the nature of the adaptive immune response (*49*).

We next explore the medium-term dynamic effect of vaccination. Figure 3D shows the ratio of the total number of primary infections during years 1.5 to 5 (i.e., after the vaccine is introduced) relative to the zero vaccination case for different values of the vaccination rate ν and the duration of vaccinal immunity 1*/*δ_vax_. Figure 3E shows the equivalent for secondary infections. The burden of primary infection decreases with increasing vaccination rate for a given value of vaccinal immunity, 1*/*δ_vax_. However, for the shortest durations of vaccinal immunity, achievable reductions in the number of secondary cases begin to plateau even for high vaccination rates. This saturation is due to the rapid return of vaccinated individuals to the partially susceptible class if vaccinal immunity is short-lived. Further, if vaccinal immunity wanes very rapidly, vaccination can transiently increase the total number of secondary cases. To further emphasize the dependence of the model results on the vaccination rate and duration of vaccinal immunity, we present time courses of infections and immunity for different durations of vaccinal immunity and vaccination rates in Fig. 3, F to I. In line with intuition, the model illustrates that both high vaccination rates and relatively long durations of vaccine-induced immunity are required to achieve the largest reductions in secondary infection burdens.

## Infection, disease, and immunity landscape for different possible futures

**Fig. 4:**
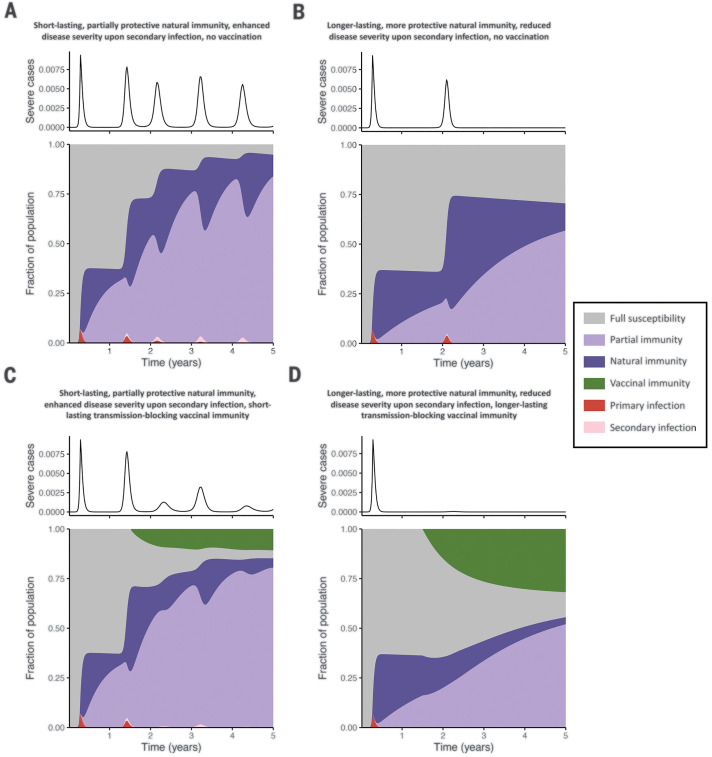
Time series of the fraction of the population with severe primary or secondary cases (top) and area plots of the fraction of the population comprising each immune (*S*_P_, *R*, *S*_S_, *V*) or infection (*I*_P_, *I*_S_) class (bottom) over a 5-year time period under four different future scenarios. In all plots, the relative transmissibility of secondary infections (α) is taken to be 1, the fraction of severe primary cases (*x*_sev,p_) is assumed to be 0.14, a seasonal transmission rate derived from the climate of New York City with no lag is assumed, and a period of social distancing during which the force of infection is reduced to 60% of its original value during weeks 16 to 55 (i.e., the scenario described in Fig. 2B) is enforced. (**A** and **B**) Two scenarios in which no vaccination occurs: a more pessimistic natural immunity scenario, with ε = 0.7, 1*/*δ = 0.5 years, and 21% of secondary cases being severe (A) and a more optimistic natural immunity scenario, with ε = 0.5, 1*/*δ = 2 years, and 7% of secondary cases being severe (B). (**C** and **D**) Two scenarios in which vaccination is introduced at a weekly rate ν of 1% at *t*_vax_ of 1.5 years after the onset of the pandemic: with all the parameters in (A) along with vaccinal immunity lasting 1*/*δ_vax_ of 0.25 years (C) or with all the same parameters as in (B) along with vaccinal immunity lasting 1*/*δ_vax_ of 1 year (D).

Figure 4 is a synoptic view of the medium-term impact of vaccination and natural immunity on the immune landscape and incidence of severe disease. We consider four scenarios, assuming seasonal transmission (as in fig. S3) and social distancing according to the pattern depicted in Fig. 2B. Figure 4, A and B, corresponds to futures without vaccination, with Fig. 4A illustrating a more pessimistic scenario of greater susceptibility to secondary infections (ε = 0.7), a relatively short period of natural immunity (1*/*δ = 0.5 years), and a greater proportion of severe cases with secondary infection. In contrast, the more optimistic future of Fig. 4B assumes reduced susceptibility to secondary infections (ε = 0.5), a longer duration of natural immunity (1*/*δ = 2 years), and a smaller proportion of severe cases with secondary infection. In both cases, the initial pandemic wave is the same, but in the more optimistic scenario (Fig. 4B), natural immunity is longer lasting and, consequently, subsequent infection peaks are delayed. Furthermore, the reduction in susceptibility to secondary infection (smaller ε) in Fig. 4B suppresses the later peaks dominated by secondary infections (Fig. 4A), and substantially less depletion of fully susceptible individuals occurs. In Fig. 4, C and D, these pessimistic and optimistic scenarios are translated into futures with vaccination, which is assumed to be introduced at a weekly rate ν of 1% after a *t*_vax_ of 1.5 years. The future described in Fig. 4C assumes all the same outcomes as in Fig. 4A and incorporates vaccination with short-lived vaccinal immunity (1*/*δ_vax_ = 0.25 years). The future presented in Fig. 4D assumes all the same outcomes as in Fig. 4B in addition to vaccinal immunity lasting for 1*/*δ_vax_ of 1 year.

Figure 4, C and D, emphasizes the important role that even an imperfect vaccine could have on SARS-CoV-2 dynamics and control [compare with (*7*, *9*, *10*)]. Vaccination substantially reduces subsequent peaks in clinically severe cases, although in the pessimistic future later infection peaks dominated by secondary infections can still occur (Fig. 4C). Furthermore, if a transmission-blocking vaccine confers a relatively long period of protection, and if we make optimistic assumptions regarding the nature of the adaptive immune response (Fig. 4D), a sufficient proportion of fully susceptible individuals can be immunized to suppress future outbreaks within the 5-year time period considered. These trends are qualitatively conserved for different vaccine deployment strategies, such as a pulse of immunization after a *t*_vax_ of 1.5 years in which a fixed percentage of the fully and partially susceptible populations (*S*_P_ and *S*_S_) are vaccinated (fig. S12). However, without sustained immunization strategies, the waning of vaccinal immunity results in a lower susceptible depletion over time and larger future outbreaks relative to the scenarios presented in Fig. 4, C and D.

## Impact of heterogeneity

### Transmission and clinical heterogeneity

COVID-19 shows marked heterogeneity in transmission and clinical severity with age and other variables (*40*). There are also marked individual heterogeneities, often associated with superspreading events. It is useful to distinguish “environmental” heterogeneity, where high transmission is associated with local environmental (or sociological) factors such as low air exchange, and “intrinsic” heterogeneity, e.g., where certain individuals have consistently higher contact rates (*40*). A number of studies have explored the possibility that intrinsically higher transmission rates for some individuals could reduce the immune threshold for natural or vaccinal herd immunity to COVID-19 (*39*, *50*), echoing classical theory (*51*).

We approximate the impact of intrinsic heterogeneity using a two-subpopulation extension of our homogeneous model (*37*) (figs. S13 to S15). As well as varying transmission between groups, the model assesses covariation between transmission rate and clinical severity. For example, this framing broadly reflects age-structured heterogeneities, in which more clinically threatened older groups might have a lower (because of fewer contacts or possible shielding) or higher [if long-term care facilities are hit (*40*)] transmission rate. We show that moderate heterogeneities do not affect our qualitative projections about the impact of partial natural or vaccinal immunity on epidemic dynamics (figs. S14 and S15). As expected, intrinsic transmission heterogeneity does reduce future burden if there is strong and durable immunity (*39*, *50*) (compare Fig. 4B with figs. S14B and S15B, particularly the subsequent epidemic peaks), because high-transmission individuals would become immune early, reducing average reproduction ratios [and modulating the herd immunity threshold (*39*, *50*)]. However, this impact of intrinsic heterogeneity is weakened [or “buffered” (*35*)] if immunity is imperfect (compare Fig. 4A with figs. S14A and S15A); this is because highly transmissive individuals (e.g., those with a larger social network) contribute proportionately more to secondary transmission when they enter the partially susceptible state. Again, this subtlety illustrates the complexities of even simple variations in immune life history.

### Vaccine hesitancy

**Fig. 5:**
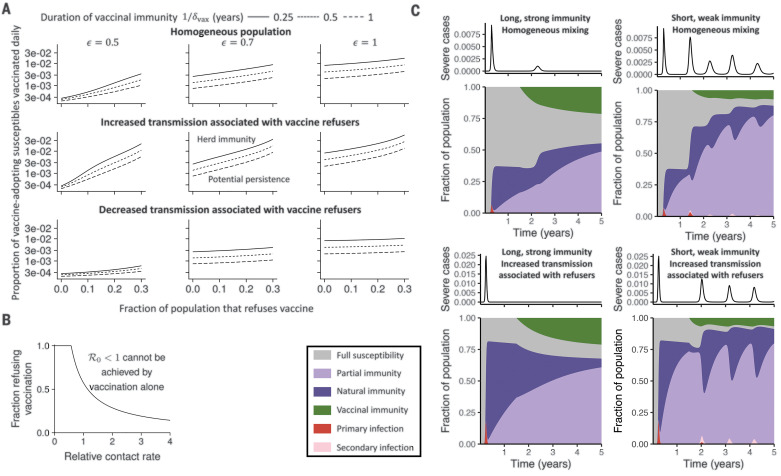
Effect of vaccine refusal on disease dynamics. (**A**) Daily proportion of vaccine-adopting individuals from the partially and fully susceptible immune classes who must be immunized in order to achieve ℛ_0_ *<* 1 as a function of the fraction of the population that refuses the vaccine (*37*) for different values of the duration of vaccinal immunity (1*/*δ_vax_ = 0.25 years, solid line; 1*/*δ_vax_ = 0.5 years, dashed line; 1*/*δ_vax_ = 1 year, dotted line) and different values of the susceptibility to secondary infection ε [ε = 0.5 (left) ε = 0.7 (middle) or ε = 1 right)]. (Top row) Homogeneous transmission between vaccine adopters and refusers (*c*_11_ = *c*_12_ = *c*_21_ = *c*_22_ = 1). (Middle row) Increased transmission associated with vaccine refusers (*c*_11_ = 1, *c*_12_ = 1.25, *c*_21_ = 1.25, and *c*_22_ = 1.5). (Bottom row) Decreased transmission associated with vaccine refusers (*c*_11_ = 1, *c*_12_ = 0.825, *c*_21_ = 0.825, and *c*_22_ = 0.75). (**B**) Maximum fraction of the population that can refuse vaccination for herd immunity to still be achieved as a function of the contact rate among vaccine refusers *c*_22_ (*37*). In (A) and (B), the transmission rate is derived from the mean value of seasonal New York City–based weekly reproduction numbers (${\bar{R}}_{0}$ = 1.75) (*37*) (fig. S2C). (**C**) Time series of the fraction of the population with severe primary or secondary cases (top) and area plots of the fraction of the population comprising each immune (*S*_P_, *R*, *S*_S_, *V*) or infection (*I*_P_, *I*_S_) class (bottom) over a 5-year time period. The parameters in the left two series are identical to those in Fig. 4C, and the parameters in the right two series are identical to those in Fig. 4D. Additionally, the fraction of the population refusing vaccines is taken to be *N*_2_ = 0.3. (Top row) Homogeneous mixing with *c*_11_ = *c*_12_ = *c*_21_ = *c*_22_ = 1. (Bottom row) Increased contacts among vaccine refusers and *c*_11_ = 1, *c*_12_ = 1.25, *c*_21_ = 1.25, and *c*_22_ = 1.5.

There is an extensive body of theory on vaccine hesitancy (*52*, *53*). In the homogeneous case, vaccine refusal essentially trades off against vaccine uptake; however, if refusers are spatially or socially clustered, there may be more impact of refusal, both epidemiologically and in terms of the social contagion which underlies it (*52*). We use a simple adaptation of our models (*37*) to explore how transmission heterogeneity influences the epidemiological impact of hesitancy (Fig. 5). As in the vaccination rate titration presented in Fig. 3, B and C, a larger fraction of vaccine refusers in a homogeneous population increases the necessary vaccination rate for herd immunity (Fig. 5A, top). This effect is amplified when the susceptibility to secondary infection is high (compare columns) or the duration of vaccinal immunity is short (compare individual curves). In the heterogeneous case, where vaccine refusers are assumed to have different transmission rates because of higher or lower adherence to NPIs, the minimum vaccination rate to achieve herd immunity is further altered. This can be seen by comparing the rows of Fig. 5A, ordered on the basis of homogeneous contact rates (top), increased contact rates for vaccine refusers (middle), and decreased contact rates for vaccine refusers (bottom). Notably, we find that when vaccine refusers have increased contact rates relative to the rest of the population, vaccination alone may not be able to prevent an outbreak (Fig. 5B). Alternatively, a decrease in contact rates for vaccine refusers decreases their impact. Finally, in Fig. 5C, we reproduce the area plots from Fig. 4, C and D, assuming that 30% of the population refuses the vaccine. This estimate is broadly consistent with recent polls conducted in the United States and Canada (*54*, *55*). We find that the overall disease burden critically depends on the duration and strength of immunity and is larger if vaccine refusers have higher contact rates relative to the rest of the population (compare top and bottom rows).

## Caveats

To focus on immune dynamics, we have made several simplifying assumptions. First, we have assumed that transmission of SARS-CoV-2 is seasonal and similar to that of the related β-CoV HCoV-HKU1, although we have also explored the effect of diminished seasonality (*37*). Second, we have simplified the important role for heterogeneities, such as age, clinical severity, transmissibility (*40*), and adaptive immune response (*16*) to primary and secondary (and beyond) infections. Notably, higher viral loads or contact rates in some individuals can lead to superspreading events and heterogeneous transmission patterns (*40*). Additionally, the severity of an infection, especially if associated with higher viremia than in mild cases, could affect the nature of the subsequent adaptive immune response, via antigen-driven expansion of the antibody response (*17*) or exhaustion of the T cell response (*18*). We have explored the effect of these heterogeneities on disease dynamics via a simple model extension (figs. S13 to S15); we find that dynamic impacts of immune variation projected by our homogeneous model are qualitatively robust to these inclusions. Finally, we have considered highly simplified scenarios for NPI adoption and vaccination.

The dynamic impact of these and other parameter variations can be explored interactively at https://grenfelllab.princeton.edu/sarscov2dynamicsplots. For example, strategies to suppress future outbreaks [e.g., (*56*)] could be simulated by increasing the duration and strength of NPIs, then exploring optimal vaccine deployment as vaccines are developed and rolled out. See (*37*) for a full discussion of all caveats and future directions.

## Conclusion

We have examined how plausible variations in the natural immune response after SARS-CoV-2 infection and vaccination could interact with seasonal drivers and NPIs to shape the medium-term epidemic dynamics, clinical burden, and immunity landscape to COVID-19. In locations where we expect substantial climatically driven seasonal variation in transmission, such as New York City, the model predicts that a reduction in susceptibility to secondary infection or a longer duration of immunity may lead to a larger secondary infection peak, which may occur earlier if the duration of natural immunity is longer. With smaller annual fluctuations in climate, we find that this nonmonotonic behavior is increasingly suppressed; however, this effect is sensitive to the assumed form of climatic influences on SARS-CoV-2 transmission, which we have taken here to be very similar to those of the related β-CoV HCoV-HKU1. The subsequent pattern of infection peaks is even more sensitive to the relative fraction and transmissibility of primary and secondary cases, as well as the fraction of severe cases for each category. Overall, whereas climatic effects or other seasonal modulators of the transmission rate increase in importance as the pandemic progresses (*25*), our results underline that understanding the immunology of secondary infection (which modulates susceptible supply) is even more dynamically important, especially in the medium term.

The pandemic trajectory can also be substantially altered by mass deployment of vaccines; however, the impact on burden is strongly dependent on the efficacy of the vaccine and the nature of the adaptive immune response. Recent vaccine trials in mice and rhesus macaques indicate the generation of robust immune responses, clinical protection from severe disease, and no evidence of ADE after viral challenge, possibly indicating a more optimistic immune scenario (*44*). Vaccine hesitancy could also decrease vaccination rates (*53*), leading to lower levels of population immunity. Nevertheless, even with imperfect vaccinal immunity and moderate vaccination rates, our results indicate that vaccination may accelerate pandemic control. Ultimately, quantitatively projecting the impact of vaccination, antivirals, and therapeutics will require more granular immuno-epidemiological models; however, parameterizing such models will continue to present huge challenges for this novel virus. We argue that a family of simple and more complex models, with a careful focus on model comparison and averaging, is the way ahead (*57*).

Our work underlines that relying on the status of infection of an individual as the main observable during an ongoing epidemic is insufficient to characterize the complex immune landscape generated by the pandemic. This is in line with ongoing calls for the development of a Global Immunological Observatory for the surveillance of population-level susceptibility and immunity to circulating pathogens, as well as the emergence of new strains (*58*–*60*). Given the increasingly recognized importance of both T cell–mediated (*14*, *15*) and antibody-mediated (*11*–*13*) adaptive immune responses in the recovery from SARS-CoV-2 infection, regular testing of antibody presence, and correlates of protection such as neutralization, as well as T cell immunity, in parallel with viral testing, will be required to adequately characterize population-level natural and vaccinal immunity to this pathogen. Specifically, our model indicates a key need to establish (i) the duration and strength of transmission-blocking and clinical immunity after primary (and subsequent) infection and vaccination; (ii) population and individual variations in these parameters (age, sex, etc.); and (iii) the impact of viral evolution, coinfection, and other pathogen characteristics on COVID-19 infection and disease. Quantifying these parameters will require long-term major investments in integrated viral and immune surveillance. Moving beyond the current pandemic, these structures (and associated developments in biology, informatics, and translation) will be powerful bases for understanding and combating inevitable future microbial threats (*58*–*60*).

This work emphasizes the complex dependence of the immune landscape generated by SARS-CoV-2 infection on the presently uncertain nature of the adaptive immune response to this virus and the efficacy of potential future vaccines. Depending on how these unfold, the model predictions for future clinical burdens range from sustained epidemics to near–case elimination. Consequently, accurately characterizing the individual immune life histories and the cumulative immune landscape of the population to SARS-CoV-2 primary and secondary infection and vaccination will be critical for the management and control of the ongoing pandemic.

## Supplementary Materials

science.sciencemag.org/content/370/6518/811/suppl/DC1 Materials and Methods Supplementary Text Figs. S1 to S15 References (*63*–*70*) MDAR Reproducibility Checklist

## Appendix

View/request a protocol for this paper from *Bio-protocol*.
